# First case of a human *Candida berthetii* systemic infection in a preterm infant: a case report

**DOI:** 10.1128/asmcr.00120-25

**Published:** 2025-11-25

**Authors:** Elaine Cristina Francisco, Fatima M. V. Porfirio, Larissa M. Favarello, Elisa J. U. Kusano, Debora D. Krenke, Regina Matielo, Denise Vilarino Louzada, Lúcio Flávio Peixoto de Lima, Lígia C. Pierrotti, Arnaldo L. Colombo

**Affiliations:** 1 Division of Infectious Diseases, Escola Paulista de Medicina-Universidade Federal de São Paulo, São Paulo, Brazil; 2 Center for Monitoring Antimicrobial Resistance Patterns and Genetic Diversity in Clinical and Environmental Isolates from Brazilian Biomes, Universidade Federal do Paraná 28122, Curitiba, Brazil; 3 Antimicrobial Resistance Institute of São Paulo (ARIES), São Paulo, Brazil.; 4 Hospital e Maternidade Sepaco, São Paulo, Brazil; 5 DASA, São Paulo, Brazil; Vanderbilt University Medical Center, Nashville, Tennessee, USA

**Keywords:** *Candida berthetii*, rare yeast pathogens, invasive fungal infections, antifungal resistance, candidemia

## Abstract

**Background:**

We report the first case of invasive human infection caused by *Candida berthetii* in a preterm infant.

**Case Summary:**

A premature neonate who required prolonged hospitalization, intensive care support, multiple surgical interventions, and broad-spectrum antibiotics developed persistent candidemia due to *C. berthetii*. Species identification was confirmed through sequencing of the internal transcribed spacer region of ribosomal DNA. Antifungal susceptibility testing, performed using the the European Committee on Antimicrobial Susceptibility Testing (EUCAST) microbroth dilution method, revealed high minimal inhibitory concentrations (MICs) to fluconazole and anidulafungin but low MICs for amphotericin B. Despite switching to liposomal amphotericin B, the infant progressed to multiple organ dysfunction and died. The poor clinical outcome was attributed to both the severity of underlying conditions associated with candidemia and the delay in starting adequate antifungal therapy.

**Conclusion:**

This case broadens the current understanding of rare yeast pathogens and highlights the critical need for improved diagnostic tools to accurately detect and identify uncommon *Candida* species. Clinicians should remain vigilant to the emergence of rare yeast pathogens causing fungemia, especially among immunosuppressed hosts undergoing invasive medical procedures and prolonged exposure to broad-spectrum antimicrobials.

## INTRODUCTION

Invasive fungal infections in neonates, particularly in premature infants with complex medical conditions, pose significant challenges in both diagnosis and treatment (1, 2). Rare and emerging *Candida* species have been increasingly associated with these infections, but there remains a lack of detailed documentation regarding their clinical impact and antifungal susceptibility (3, 4). *Candida berthetii* (Boidin, Pignal, Mermiér & Arpin), first described in 1963 from an isolate found in gum arabic from a forest tree, was later identified in the ambrosia beetle *Xyleborus volvulus* (5, 6). Notably, this species has not been documented in wildlife animals or humans, highlighting challenges in understanding its ecological niche and its potential role in human infections. This study describes the first case of human systemic infection caused by *C. berthetii* in a premature neonate, who developed a persistent bloodstream infection despite antifungal therapy.

## CASE PRESENTATION

A preterm male neonate, the first of a twin pregnancy, was transferred to the neonatal intensive care unit (NICU) of a tertiary care center with 6 days of life for the surgical management of hypoplastic left heart syndrome (HLHS). The infant was born at 28 weeks via an emergency cesarean section due to preterm labor triggered by congenital heart disease, with a birth weight of 1,610 g and Apgar scores of 7 and 6 at 1 and 5 min, respectively. Intrauterine diagnosis of HLHS was established by fetal echocardiography.

During the first 7 days of NICU admission, the infant remained intubated and received empirical broad-spectrum antibiotics to treat a putative clinical event of sepsis with all cultures negative. Extubation was possible at 12 days of life, followed by support with non-invasive positive pressure ventilation. Total parenteral nutrition (TPN) and enteral feeding were initiated, targeting adequate weight gain for surgical intervention.

On day 16 of life, the patient developed low cardiac output syndrome secondary to imbalance between pulmonary and systemic blood flow (Qp:Qs), which was complicated by necrotizing enterocolitis (NEC). Management of NEC included bowel rest, broad-spectrum antibiotics, and reintubation. Two days later, pneumoperitoneum was diagnosed, and a percutaneous abdominal drain was placed at the bedside. At that time, antimicrobial therapy included meropenem (40 mg/kg/day), daptomycin (6 mg/kg/day), and fluconazole (12 mg/kg/day). The antifungal was added to the empirical antimicrobial regimen for NEC due to the presence of risk factors for *Candida* infection and was maintained for 37 days.

Surgical correction of HLHS was postponed due to the clinical instability of the neonate. At 25 days of life, the patient underwent ductal stent placement and bilateral pulmonary artery banding to optimize systemic perfusion. Postoperatively, the patient developed acute kidney injury (KDIGO stage 2), significant fluid overload (>20% above baseline), and was managed with diuretics, albumin, and mannitol. Due to low body weight and contraindications, renal replacement therapy was not pursued. The patient gradually improved.

Due to clinical instability, intestinal perforation caused by NEC was initially managed conservatively with peritoneal drainage. Following clinical improvement, corrective surgery was performed at 39 days of life (exploratory laparotomy, lysis of adhesions, jejunostomy at 40 cm distal to the ligament of Treitz, multiple enterectomies, and primary jejunal anastomosis). At 47 days, he underwent a balloon atrial septostomy (Raskind procedure) for restrictive atrial septal defect.

The patient continued under intensive care, and at 3 months of age, the infant developed signs and laboratory findings suggestive of bloodstream infection, including hypothermia, hypotension, and thrombocytopenia (67,000/mm^3^, reference value:150,000–450,000/mm^3^), and elevated C-reactive protein (CRP) (13.23 mg/dL, reference value: <0.3 mg/dL). Blood cultures were obtained via central venous catheter and processed using BD BACTEC (Becton Dickinson, NJ, USA) for automatic detection. Preliminary identification of the blood stream pathogen was obtained by MALDI-TOF-MS (VITEK MS V.3.2, BioMérieux, Marcy l'Etoile, France) which reported *Candida* spp. with low confidence level to species identification. Fluconazole therapy was initiated immediately. However, despite 30 days of antifungal treatment and intensive care support, the infant remained clinically unstable. Along this period, a total of eight cultures of different clinical samples (blood, catheter tips, and peritoneal fluid) were all positive for *Candida* spp.

Considering the lack of accurate identification of the yeast pathogens and the persistent clinical instability of the infant, all isolates were sent to Laboratório Especial de Micologia (LEMI)-Federal University of Sao Paulo, a reference laboratory for fungal identification and antifungal susceptibility in Brazil. Species identification of all isolates was performed by sequencing the ITS1–5.8S-ITS2 rDNA region (7, 8). Sequences were aligned and compared with the reference sequences in the NCBI database using BLASTn (http://ncbi.nlm.nih.gov). Sequences with a percentage of identity and sequence coverage of ≥98% and an E-value of <10^−5^ were considered (8).

Antifungal susceptibility testing was performed according to the EUCAST E.Def 7.4 method (9) against the following antifungal agents: fluconazole, voriconazole, anidulafungin, and amphotericin B (AMB) (Sigma-Aldrich, St. Louis, MO, USA). Minimal inhibitory concentration (MIC) readings were performed spectrophotometrically after 24 h of incubation, at 35 ± 2°C, using a 50% inhibition threshold for azoles and echinocandins, and 100% inhibition for AMB.

The ITS-sequencing results identified all isolates as *C. berthetii*, a species not previously associated with human infection. Eight isolates from blood, peritoneal fluid, and catheter tips were recovered at distinct time points throughout the course of infection, and all of them were identified as *C. berthetti*. All isolates displayed elevated MICs to fluconazole (>64 µg/mL), voriconazole (1–2 µg/mL), and anidulafungin (1 µg/mL), with low MIC to AMB (MIC = 0.25 µg/mL) (Table 1). Antifungal therapy was switched to liposomal amphotericin B. Nevertheless, the patient’s condition continued to deteriorate, progressing to multiple organ dysfunction and dying 5 days later despite therapy with AMB along this period.

**TABLE 1:** *In vitro* antifungal susceptibility of eight *Candida berthetii* clinical isolates

Isolate	Sampling site	Sampling date	MIC (µg/mL) of:			
			Fluconazole	Voriconazole	Anidulafungin	Amphotericin B
2078/2021	Peritoneal fluid	05/04/2021	64	2	1	0.25
2079/2021	Blood	05/06/2021	64	2	1	0.25
2080/2021	Blood	05/06/2021	64	2	1	0.25
2120/2021	Blood	05/06/2021	64	1	1	0.25
2121/2021	Blood	05/25/2011	64	1	1	0.25
2122/2021	Proximal catheter tip	05/06/2021	64	1	1	0.25
2123/2021	Distal catheter tip	05/27/2021	64	1	1	0.25
2124/2021	Blood	05/31/2021	32	0.5	1	0.25

Due to the rarity of *C. berthetti* isolation in BSIs, environmental surveillance was performed by the local hospital infection control team, but no single culture was positive across all samples collected from hospital surfaces, intravenous solutions, and medicines. *C. berthetii* was only identified in the clinical samples.

## DISCUSSION

This report represents the first documented instance of *C. berthetii* causing invasive infection in a human being in the setting of immunosuppression and long-term exposure to intensive care support. Previously, this species had been scarcely cultured from a few environmental samples collected from *Acacia* gum and an insect called *Xyleborus volvulus* (5, 6).

In the present case, systemic infection was confirmed by the accurate identification of the pathogen across eight cultures collected at different time points from multiple clinical specimens, including four blood samples. Identification was achieved via sequencing of the ITS region of rDNA, which provided high-resolution species-level assignment. Based on this method, all isolates were unequivocally identified as *C. berthetii*, exhibiting 100% sequence identity and coverage compared with the reference strain *C. berthetii* CBS 6113. As expected, MALDI-TOF MS commercial database failed to identify this pathogen, highlighting the need for updates to available libraries for the detection of rare yeast species, such as *C. berthetii* (10).

High MICs for fluconazole and anidulafungin were obtained using standardized broth microdilution method according to EUCAST protocol. Rare yeast pathogens, such as *Candida berthetii*, often express elevated MICs for at least one class of antifungal agents (4, 11). Infections caused by these rare pathogens are particularly challenging to manage due to the limited clinical data available in the literature and the lack of susceptibility breakpoints to interpret antifungal susceptibility tests (11, 12). The persistence of candidemia despite antifungal treatment is consistent with *in vitro* data, suggesting low susceptibility of *C. berthetii* to the antifungal used. These findings underscore the critical need for precise species identification and comprehensive antifungal susceptibility testing, particularly in the context of refractory infections caused by rare and emerging yeast pathogens.

Preterm neonates are uniquely predisposed to invasive infections caused by a large spectrum of pathogens, including rare yeasts (13). The immunological immaturity and low weight at birth of preterm infants, prolonged hospitalization and intensive care support, exposure to broad-spectrum antibiotics and multiple invasive medical procedures are all conditions that increase the risk of preterm neonates to acquire infections by emerging yeast pathogens (2, 14–18). All these risk conditions were present in our present case of systemic infection due to *C. berthetii*.

The poor prognosis observed in the present infant case was likely attributable to the severity of the underlying comorbidities associated with candidemia, the inherent immunological immaturity of preterm neonates, and delays in both the accurate pathogen identification and the initiation of appropriate antifungal therapy. This case broadens the current understanding of rare yeast pathogens and also underscores the critical need for improved diagnostic tools to accurately detect uncommon *Candida* species. Early recognition and tailored treatment are essential, particularly in immunocompromised patients undergoing invasive medical procedures and exposed to broad-spectrum antibiotics. Clinicians should remain vigilant to the emergence of rare yeast pathogens causing fungemia, particularly in immunocompromised patients undergoing multiple invasive medical procedures and receiving prolonged broad-spectrum antibiotics (13, 14). Further investigation is warranted to elucidate the eco-epidemiology, virulence factors, and mechanisms of antifungal resistance of *C. berthetii*.

## Data Availability

The genomic data have been deposited in GenBank NCBI repositories under the following accession numbers: PV753053, PV753054, PV753055, PV753056, PV753057, PV753058, PV753059, and PV753060.

## References

[1] Chen YN, Hsu JF, Chu SM, Lai MY, Lin C, Huang HR, Yang PH, Chiang MC, Tsai MH. 2022. Clinical and microbiological characteristics of neonates with candidemia and impacts of therapeutic strategies on the outcomes. J Fungi (Basel) 8:465. doi:10.3390/jof8050465 35628721 PMC9148079

[2] Bal AM, Pana ZD, Carlesse F, Marek A, Seidel D, Mehler K, Butzer S, Sprute R, Stemler J, Ludwig-Bettin D, Groll AH, Cornely OA, Mellinghoff SC. 2025. The Paediatric European Confederation of Medical Mycology (ECMM) quality (Paed-EQUAL) Candida score for the management of candidemia in children and neonates. Mycoses 68:e70041. doi:10.1111/myc.70041 40071950

[3] Tsai MH, Hsu JF, Yang LY, Pan YB, Lai MY, Chu SM, Huang HR, Chiang MC, Fu RH, Lu JJ. 2018. Candidemia due to uncommon Candida species in children: new threat and impacts on outcomes. Sci Rep 8:15239. doi:10.1038/s41598-018-33662-x 30323257 PMC6189077

[4] Kumar S, Kumar A, Roudbary M, Mohammadi R, Černáková L, Rodrigues CF. 2022. Overview on the infections related to rare Candida species. Pathogens 11:963. doi:10.3390/pathogens11090963 36145394 PMC9505029

[5] Boidin J, Pignal MC, Mermier F, Arpin M. 1963. Quelques levures camerounaises. Cahiers de la Maboké 1:86–100.

[6] Cruz LF, Menocal O, Mantilla J, Ibarra-Juarez LA, Carrillo D. 2019. Xyleborus volvulus (Coleoptera: Curculionidae): biology and fungal associates. Appl Environ Microbiol 85:e01190-19. doi:10.1128/AEM.01190-19 31375485 PMC6752025

[7] Ende AHG, Hoog GS. 1999. Variability and molecular diagnostics of the neurotropic species Cladophialophora bantiana. Stud Mycol 43:151–162.

[8] Merseguel KB, Nishikaku AS, Rodrigues AM, Padovan AC, e Ferreira RC, de Azevedo Melo AS, Briones M da S, Colombo AL. 2015. Genetic diversity of medically important and emerging Candida species causing invasive infection. BMC Infect Dis 15:57. doi:10.1186/s12879-015-0793-3 25887032 PMC4339437

[9] EUCAST. 2023. EUCAST definitive document EUCAST E.Def 7.4 method for the determination of broth dilution minimum inhibitory concentrations of antifungal agents for yeasts. Available from: https://www.eucast.org/fileadmin/src/media/PDFs/EUCAST_files/AFST/Files/EUCAST_E.Def_7.4_Yeast_definitive_revised_2023.pdf

[10] Robert MG, Cornet M, Hennebique A, Rasamoelina T, Caspar Y, Pondérand L, Bidart M, Durand H, Jacquet M, Garnaud C, Maubon D. 2021. MALDI-TOF MS in a medical mycology laboratory: on stage and backstage. Microorganisms 9:1283. doi:10.3390/microorganisms9061283 34204665 PMC8231132

[11] Pérez-Hansen A, Lass-Flörl C, Lackner M, Rare Yeast Study Group. 2019. Antifungal susceptibility profiles of rare ascomycetous yeasts. J Antimicrob Chemother 74:2649–2656. doi:10.1093/jac/dkz231 31203366

[12] Arendrup MC, Boekhout T, Akova M, Meis JF, Cornely OA, Lortholary O, European Society of Clinical Microbiology and Infectious Diseases Fungal Infection Study Group, European Confederation of Medical Mycology. 2014. ESCMID and ECMM joint clinical guidelines for the diagnosis and management of rare invasive yeast infections. Clin Microbiol Infect 20:76–98. doi:10.1111/1469-0691.12360 24102785

[13] Cook A, Ferreras-Antolin L, Adhisivam B, Ballot D, Berkley JA, Bernaschi P, Carvalheiro CG, Chaikittisuk N, Chen Y, Chibabhai V, et al.. 2023. Neonatal invasive candidiasis in low- and middle-income countries: data from the NeoOBS study. Med Mycol 61:myad010. doi:10.1093/mmy/myad010 36881725 PMC10026246

[14] Zhang X, Zhivaki D, Lo-Man R. 2017. Unique aspects of the perinatal immune system. Nat Rev Immunol 17:495–507. doi:10.1038/nri.2017.54 28627520

[15] Raymond SL, Stortz JA, Mira JC, Larson SD, Wynn JL, Moldawer LL. 2017. Immunological defects in neonatal sepsis and potential therapeutic approaches. Front Pediatr 5:14. doi:10.3389/fped.2017.00014 28224121 PMC5293815

[16] Parra-Llorca A, Pinilla-Gonzlez A, Torrejón-Rodríguez L, Lara-Cantón I, Kuligowski J, Collado MC, Gormaz M, Aguar M, Vento M, Serna E, Cernada M. 2023. Effects of sepsis on immune response, microbiome and oxidative metabolism in preterm infants. Children (Basel) 10:602. doi:10.3390/children10030602 36980160 PMC10046958

[17] Hibbert JE, Currie A, Strunk T. 2018. Sepsis-induced immunosuppression in neonates. Front Pediatr 6:357. doi:10.3389/fped.2018.00357 30555806 PMC6281766

[18] Sharma M, Chakrabarti A. 2023. Candidiasis and other emerging yeasts. Curr Fungal Infect Rep 17:15–24. doi:10.1007/s12281-023-00455-3 36741271 PMC9886541

